# Primary Iliopsoas Abscess and Drug-Induced Liver Injury in the Emergency Department: A Case Report

**DOI:** 10.3390/diseases12120326

**Published:** 2024-12-12

**Authors:** Ovidiu Alexandru Mederle, Laurentiu Sima, Daian Ionel Popa, Carmen Gabriela Williams, Diana Mitu, Dumitru Șutoi, Cosmin Iosif Trebuian, Mircea Selaru, Dan Lolos, Ana-Maria Pah, Florina Buleu

**Affiliations:** 1 Department of Surgery, Emergency Discipline, “Victor Babes” University of Medicine and Pharmacy, 300041 Timisoara, Romania; mederle.ovidiu@umft.ro (O.A.M.); dumitru.sutoi@umft.ro (D.Ș.); trebuian.cosmin@umft.ro (C.I.T.); 2 Emergency Municipal Clinical Hospital, 300254 Timisoara, Romania; drcarmen.williams@yahoo.com (C.G.W.); diana-alexandra.mitu@umft.ro (D.M.); 3 Department of Surgery I, “Victor Babes” University of Medicine and Pharmacy, 300041 Timisoara, Romania; 4 Department of Doctoral Studies, “Victor Babes” University of Medicine and Pharmacy, E. Murgu Square No. 2, 300041 Timisoara, Romania; daian-ionel.popa@umft.ro; 5 Department of General Surgery, “Victor Babes” University of Medicine and Pharmacy, 300041 Timisoara, Romania; selaru.mircea@umft.ro; 6 Faculty of Dental Medicine, “Victor Babeș” University of Medicine and Pharmacy Timișoara, Eftimie Murgu Sq. No. 2, 300041 Timișoara, Romania; lolosdan@umft.ro; 7 Department of Cardiology, “Victor Babes” University of Medicine and Pharmacy, E. Murgu Square No. 2, 300041 Timisoara, Romania; anamaria.pah@umft.ro (A.-M.P.); florina.buleu@umft.ro (F.B.)

**Keywords:** iliopsoas abscess, surgical drainage, emergency department, multidisciplinary approach, drug-induced liver injury, critical care, personalized medicine

## Abstract

Background and objective: Iliopsoas abscess (IPA) is a rare condition with varied symptomology and etiology. Less than one-third of patients with IPA present in the emergency department (ED) with the traditional triad of fever, back pain, and restricted hip motion (or limp), leading to delays in diagnosis and management. Acute liver failure is also a rare clinical presentation in the ED, being associated with high morbidity and mortality. It occurs most often in young patients without pre-existing liver disease, presenting unique challenges in clinical management. Most cases currently happen because of drug-induced liver injury (DILI), mainly from acetaminophen or idiosyncratic drug reactions. This case report aims to raise awareness among healthcare professionals regarding the two atypical presentations in ED and introduce a potential differential diagnosis when evaluating patients with fever and back pain or liver enzyme elevations with or without nonspecific symptoms associated with the development of jaundice. The intention is to provide insights into the signs and symptoms that may indicate the presence of an iliopsoas abscess and prompt additional investigations. Case report: Here, we describe a case of primary iliopsoas abscess associated with drug-induced liver injury in our ED. The patient complained of pain in the left lumbar region and fatigue that started two weeks before this presentation, claiming that, during the previous night, the pain suddenly worsened. At the first clinical examination in the ED, the patient presented pain at palpation in the right hypochondriac and left lumbar regions, accompanied by fever, vomiting, and jaundice. On abdominal ultrasonography, the diagnosis of acute cholangitis was suspected. The laboratory test shows leukocytosis with neutrophilia, thrombocytosis, elevated liver enzymes, and hyperbilirubinemia with the predominance of indirect bilirubin. After analyzing the laboratory test results, we repeated and performed a more detailed anamnesis and medical history of the patient. Because of her increasing pain and persistent fever, she recognized excessive consumption in the last five days of drug-induced hepatotoxicity. We performed abdominal and pelvic computed tomography, which confirmed the diagnosis of cholelithiasis observed with the diameter of the bile duct within normal limits but also showed an abscess collection fused to the interfibrillar level of the left iliopsoas muscle, a diagnosis we most likely would have missed. The patient was hospitalized in the General Surgery Department, and surgical abscess drainage was performed. The patient’s evolution was excellent; she was discharged after 11 days. Conclusions: The case presented here exemplifies how iliopsoas abscess, a rare cause of back pain, can quickly go unrecognized, especially in the emergency department. Our experiences will raise awareness among doctors in emergency departments about this uncommon but essential diagnosis. With advancements in diagnostic tools and techniques, we hope that more cases of iliopsoas abscess will be accurately diagnosed. Moreover, no case report from the literature has presented IPA associated with DILI. This case is unique because our patient did not exhibit classic features of either pathology. This case also emphasizes the importance of a medical history that includes thorough evaluations of potential high utilization of drug-induced hepatotoxicity.

## 1. Background

Mynter et al. documented the occurrence of an iliopsoas abscess (IPA), a suppurative collection in the compartment of the psoas and iliacus muscles, in 1881 [1]. It is a rare condition, with a global reported incidence between 0.4 and 12/100,000 in early studies, but the actual incidence is unknown [2,3,4].

There are two classifications for iliopsoas abscesses based on their origin: primary and secondary. Primary abscesses occur when an infection from a distant site spreads through the bloodstream or lymphatic system. A single organism typically causes these abscesses, which may be hidden. On the other hand, secondary abscesses result from the direct extension of infection from nearby structures such as the gastrointestinal or genitourinary tract, lumbar spine, or hip region. It has been reported that secondary iliopsoas abscesses have a higher mortality rate than primary ones [4].

The symptoms of iliopsoas abscess can be unclear and diverse. The traditional triad of fever, back pain, and restricted hip movement (or limp), known as the psoas-muscle sign, is only observed in 30% of cases, making diagnosis challenging [1,5]. Consequently, the condition is often missed during the initial visit to the emergency department [5].

Another rare clinical presentation in the emergency department (ED) associated with high morbidity and mortality is acute liver failure. This occurs most often in young patients without pre-existing liver disease, presenting unique challenges in clinical management [6]. Currently, most cases occur because of drug-induced liver injury, especially acetaminophen or idiosyncratic drug reactions [7].

Previous reports need to adequately discuss the diagnostic complexities associated with iliopsoas abscess, mainly when it refers to emergency physicians. Therefore, this report aims to share our firsthand experience in our emergency department diagnosing these uncommon but important diseases, IPA and DILI.

## 2. Case Report

A 49-year-old female patient presented to our Emergency Department with complaints of pain in the lower left abdomen, specifically in the left iliac fossa and right hypochondriac region, accompanied by fever and vomiting. Upon physical examination, the patient exhibited normal breathing (16 breaths per minute) and was stable regarding hemodynamics. However, she had jaundiced skin and sclera (without knowing the exact duration of the jaundice, the patient claimed that she did not notice being jaundiced), with a blood pressure reading of 130/80 mmHg, a heart rate of 80 beats per minute, a body temperature of 38.8 °C, a Glasgow Coma Score (GCS) of 15/15, and a peripheral oxygen saturation (SpO_2_) of 97% while breathing ambient air.

The patient complained of pain in the left lumbar region and fatigue that started two weeks before this presentation, claiming that, during the previous night, the pain suddenly worsened. From her medical history, she mentioned essential hypertension under treatment and untreated hypercholesterolemia. Regarding living and working conditions, the patient lives in the country with her family as a housewife. Also, the patient denies vicious behaviors such as smoking, alcohol, or drugs.

**Figure 1:**
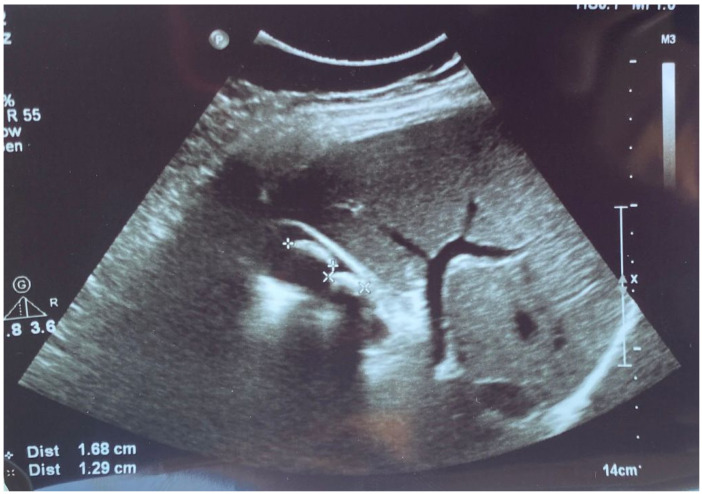
Abdominal ultrasound images of the gallbladder showed lithiasis (two calculi of 1.68 cm and 1.29 cm) associated with acoustic shadows in the transverse plane.

The patient performed an abdominal ultrasound, which revealed multiple gallstones, but the central bile duct was in a normal range value (Figure 1), so acute cholangitis was initially suspected.

Blood was taken for lab tests, and she was scheduled for an abdominal and pelvis computed tomography (CT) scan with a contrast substance. She received an intravenous perfusion with 1 g acetaminophen, 40 mg pantoprazole, 40 mg drotaverine, and 500 mL normal saline (0.9%) solution, but she remained unresponsive to the administration of the antalgic and antispastic treatment. Until the CT was performed, the blood tests arrived, which were severely increased (Table 1).

The patient’s laboratory analyses showed leukocytosis with neutrophilia, reactive thrombocytosis associated with infection, hepatic cytolysis with increased ALT (alanine aminotransferase) and AST (aspartate aminotransferase), hyperbilirubinemia with a predominance of indirect bilirubin, and normal urinalysis results.

**Figure 2:**
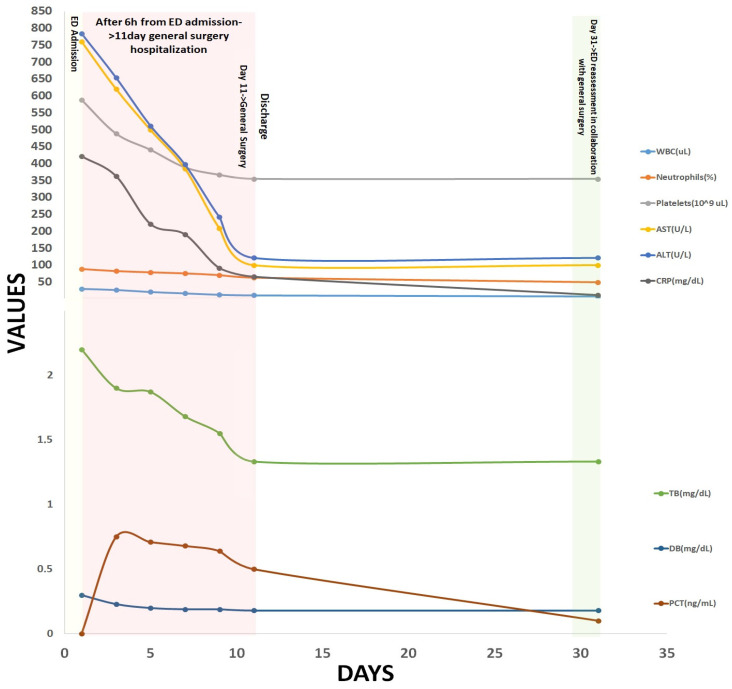
Laboratory test values evolved from ED admission until reassessment (day 31) in the ED in collaboration with our general surgery department.

After analyzing the laboratory test results, we repeated and created a more detailed medical history and anamnesis of the patient. As a result of increasing pain, the patient had taken several medications over the last five days, including acetaminophen, naproxen, ketoprofen, metamizole, amoxicillin + clavulanic acid, diclofenac, and ibuprofen. However, she could not provide us with the exact dosage of these medications but mentioned that she had consumed a significant number of pills. Based on these findings, we also considered the diagnosis of drug-induced liver injury (Figure 2).

**Figure 3:**
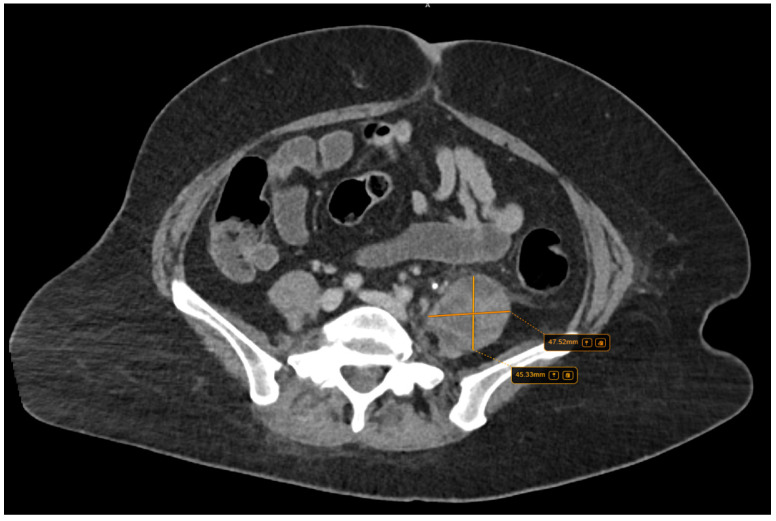
Collections of fused abscesses at the interfibrillar level of the left psoas muscle that cause volumetric growth (4.53/4.75 cm) compared to the contralateral side and associate densifications in the adjacent fatty tissue.

Further, the abdominal and pelvis CT scan with contrast substance showed the cholelithiasis observed on abdominal ultrasound with common bile duct diameter in normal limit values and fused abscess-type collections at the interfibrillar level of the left iliopsoas muscle. The CT also shows hepatomegaly with cranio-caudal dimensions of the right hepatic lobe of 220 mm, a clear, regular outline, a homogeneous structure, and a cholecyst with multiple calculi up to 17 mm (Figure 3).

In the emergency department, she received intravenous fluid resuscitation, metamizole 500 mg, drotaverine 80 mg, and an empiric antibiotic (ceftriaxone). Based on the following table (Table 1), where we present a short literature review of the documented origins of secondary spread to the psoas muscles, we decided to take additional chest radiography and blood tests in the ED to identify the cause of the patient’s left iliopsoas abscess.

**Figure 4:**
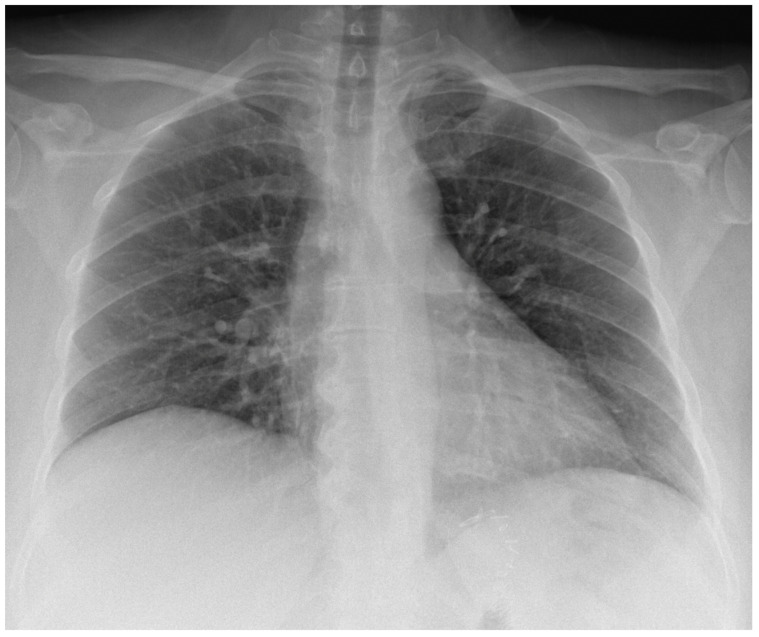
The chest radiography was performed in the ED.

The chest radiography showed clear lungs and pleural spaces and regular cardio mediastinal counter (Figure 4), thus excluding tuberculosis and, implicitly, Pott’s disease and tuberculous spondylitis as causes of iliopsoas abscess. Despite a thorough CT scan examination, no evidence of colonic micro-perforations was found, leaving the cause uncertain. Additionally, the patient explicitly denied any past involvement in intravenous drug abuse. Furthermore, we requested an evaluation of the patient by the general surgeon on call, who recommended emergency hospitalization and surgical drainage, so she was transferred to the general surgery department.

**Figure 5:**
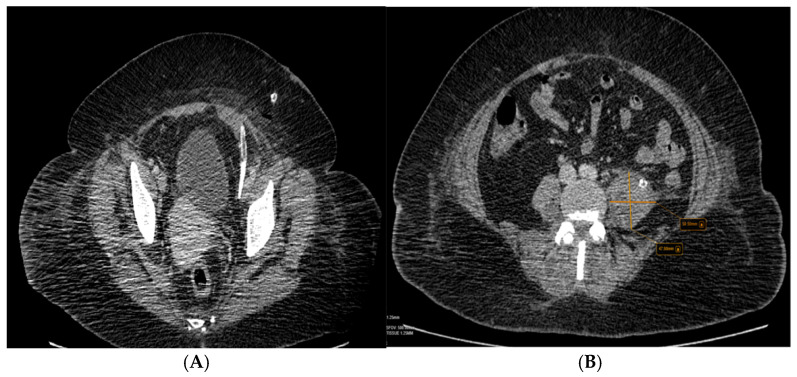
(**A**,**B**) The second abdominal and pelvis CT scan with contrast substance was performed after surgical drainage, which can be seen in the drain tubes.

After the preoperative preparation, surgical intervention is performed, and the Dos Santos incision is practiced. After dissection of the anatomical planes, a voluminous abscess is drained from the level of the left psoas muscle, taking cultures from this level simultaneously. It continues with the debridement and washing of the operative area, placing drain tubes at this level, one with the tip located in the left paravesical pelvis (Figure 5A) and one with an ascending trajectory along the left iliopsoas muscle (Figure 5B) and the tip on its ventral side, next to the L3 vertebral body, as can be seen in the second abdominal and pelvis CT scan with contrast substance performed after surgical drainage. The drain tubes were removed after nine days. The primary intention was chosen from the perspective of wound healing. The abscess was treated surgically with incision and drainage, and the wound was relatively clean. The wound’s edges were brought together and healed with minimal scarring.

The fluid culture from the iliopsoas abscess was positive for methicillin-sensitive Staphylococcus aureus (MSSA). According to the antibiogram, the identified germ was sensitive to Linezolid. Thus, it has been decided that treatment the subject should be started with Linezolid 600 mg twice a day for ten days. During hospitalization, the patient was screened for possible sources of infection. Blood and urine cultures were normal, as well as the hepatitis A, B, C, and HIV infection. She underwent a gastroscopy and colonoscopy with normal results. Figure 2 depicts the patient’s laboratory test values during all our monitoring, including hospitalization, demonstrating the favorable postoperative evaluation, with a slight decrease in blood test values, including total bilirubin and liver enzymes. After 11 days of hospitalization in the general surgery department, she was discharged in good general condition, appetizing, and afebrile.

**Figure 6:**
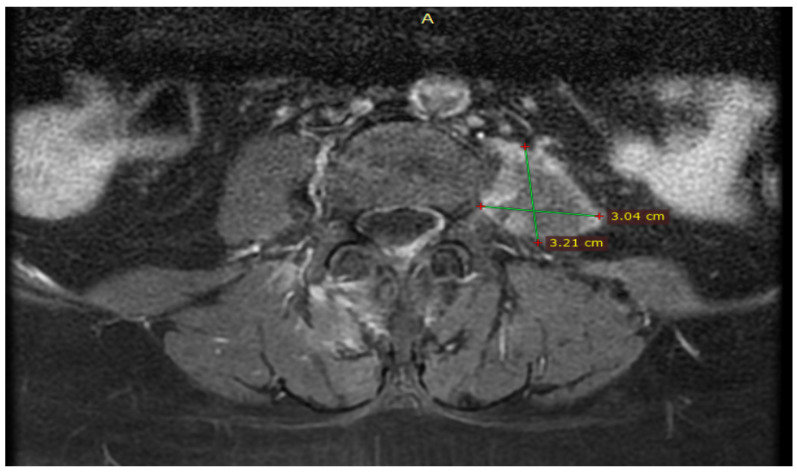
MRI of the thoracic and lumbar spine performed at 31 days reassessment showed evacuated abscess of the left iliopsoas muscle with minimal residual muscle inflammation.

During hospitalization, she was advised to have an elective laparoscopic cholecystectomy, which she declined. Because endoscopic retrograde cholangiopancreatography (ERCP) or magnetic resonance cholangiopancreatography (MRCP) cannot be performed in our hospital, she was offered to have these investigations performed in our primary county hospital, but she also delayed these investigations. At the reassessment after 31 days, the patient stated that she did not undergo any other ultrasound or blood test during this period due to the remission of symptoms and jaundice. She was again medically and clinically reevaluated in the Emergency Department in collaboration with the general surgeon, where no sign of infection was observed, and laboratory tests were in the normal range. Also, the magnetic resonance imaging (MRI) of the thoracic and lumbar spine performed in the 31 days did not identify a secondary cause for her left iliopsoas abscess (Figure 6).

## 3. Discussion

Because of its variable and nonspecific presentations and its rare occurrence in the emergency department, the diagnosis of iliopsoas abscess may be delayed or misdiagnosed [24]. The diagnostic challenges of psoas abscess, particularly in the context of emergency physicians, have yet to be adequately addressed in literature reports. In an early study conducted by Chen et al., they documented a 1-year experience with the diagnosis of iliopsoas abscess in 10 patients admitted to the ED. Of these ten patients, only seven were diagnosed in the ED, while the remaining three were initially admitted with this diagnosis. Among those seven patients, their initial ED diagnoses varied, including fever of unknown origin (2 patients), septic shock (2 patients), shock (1 patient), sepsis (1 patient), and peritonitis (1 patient). The most commonly reported symptom was pain, with 80% of patients experiencing it and 50% explicitly complaining of back pain. The classic triad of fever, back pain, and limp, typically associated with iliopsoas abscess, was only present in 30% of patients [5]. Over two years, a total of 32 patients who were admitted to a university hospital were also analyzed. The classic triad of IPA was only detected in 13 patients (40.6%). Of the 32 patients, 18 (56.3%) had primary IPA, while 14 (43.7%) had secondary IPA [25]. In a multicentric study conducted by Lee et al., it was discovered that IPA originated primarily in 50 patients (28.4%) and secondarily in 126 patients (71.6%). This finding was only recently reported [26].

As reported in various other studies, Staphylococcus aureus is the most common causative organism of primary IPA, and it was also the cause of the iliopsoas abscess in our patient.

The patient we managed at ED presented with iliopsoas abscess symptoms, fever, and discomfort in the left side of her back, which started 14 days before she arrived at our emergency department. Nonetheless, in the case of our patient, although she had performed various investigations and clinical examinations, no secondary cause was found. Her left iliopsoas abscess was successfully detected through a CT scan and effectively managed using surgical drainage and intravenous antibiotics. This case emphasizes the importance of maintaining a high level of suspicion concerning identifying this condition promptly, as misdiagnosis may occur. In addition, it is essential to note that not all cases of iliopsoas abscess present with the traditional triad of symptoms [1]. If acute cholangitis had not been coincidentally suspected on abdominal ultrasound and the subsequent abdominal and pelvic CT scan performed, the diagnosis of iliopsoas abscess in our patient might have been entirely missed during a routine examination in the emergency department.

The identification of IPA is strongly dependent on imaging methods, with computed tomography being considered the gold standard due to its exceptional accuracy [27]. Alternative techniques, such as magnetic resonance imaging and ultrasound, also present opportunities for early detection. Additionally, point-of-care ultrasound (POCUS) can be instrumental in managing these cases within the Emergency Department [28,29].

On the other hand, the successful outcome of our patient’s treatment aligns with the existing literature on the selection of empiric antibiotics in emergencies and surgical drainage of abscesses [8,30], even if the literature suggests that open surgical drainage should be pursued when percutaneous drainage is ineffective or contraindicated, particularly in situations where the abdominal issue is accompanied by another surgical condition, like Crohn’s disease, or when dealing with multiloculated abscesses [31]. Scientific research indicates that open surgical drainage often has better results in achieving complete drainage than percutaneous drainage methods [32]. The death rates associated with surgical and percutaneous drainage of IPAs are 4% and 2%, respectively [33]. The general surgeon decided to perform open surgical drainage in our patient, although image-guided percutaneous ultrasound and CT could also have been carried out.

Our patient is also experiencing associated DILI. To enhance the prediction of a drug’s potential to induce liver injury and the severity of that injury, a DILI score model has been created, taking into account factors such as drug lipophilicity (log P ≥ 3), the covalent binding of reactive metabolites, and a daily oral dosage of the drug (≥100 mg) [34]. It is crucial to acknowledge that DILI is conventionally divided into two categories: intrinsic (or direct) and idiosyncratic. Typically, intrinsic DILI correlates with dosage, affecting a significant percentage of individuals who have been exposed to the drug, making it predictable, with onset occurring within a brief timeframe of hours to days. In contrast, idiosyncratic DILI is generally not associated with dosage, although a threshold of 50–100 mg/day is often necessary [35]. However, in this case, the patient is using several medications, including acetaminophen, naproxen, ketoprofen, metamizole, amoxicillin + clavulanic acid, diclofenac, and ibuprofen, all under various trade names, without a clear specification of the precise daily dosage. These active ingredients are also acknowledged in DILI guidelines as substances known to induce hepatotoxicity [36]. Acetaminophen is the archetypal drug responsible for intrinsic DILI, contributing to half of these cases progressing to acute liver failure in both North America and Europe, a drug that also our patient has been taking in the last five days under various trade names. In addition, half of these cases are caused by single overdoses. In contrast, the other half are unintentional, usually occurring following multiple-day paracetamol use at daily doses of 4 to 10 g/day, although several cases have also been documented at doses of 2 to 4 g/day [35]. The hepatotoxicity associated with amoxicillin-clavulanic acid has been observed to show a greater prevalence of cholestatic lesion types. Yet, the factors influencing its clinical manifestation remain unclear. A study examining the DILI registry in Spain—one of the few nations globally to maintain such a registry—revealed that amoxicillin-clavulanic acid-related hepatotoxicity was documented in 69 patients (36 men; average age 56 years), accounting for 14% of all reported hepatotoxicity cases in the Registry. Among the cases documented in this registry, age emerged as the critical factor influencing the biochemical manifestation of hepatotoxicity due to amoxicillin-clavulanic acid. Younger patients exhibited cytolytic lesions and experienced shorter treatment durations, while older individuals showed cholestatic or mixed-type lesions associated with extended therapy using amoxicillin-clavulanic acid [37].

Although our patient was taking several drugs that induced hepatotoxicity, we calculated, even in this case, the causality of drug-induced hepatotoxicity by applying the Roussel Uclaf Causality Assessment Method/Council for International Organizations of Medical Sciences Causality Assessment Method score [38] as follows: DILI mixed type (hepatocellular and cholestatic), onset between 5 and 90 days (+2), time from drug withdrawal to the onset of reaction <15 days (+1), with the risk factors being alcohol (0), age <55 years (0), decrease in total bilirubin >/= 50% in 180 days (+2), concomitant therapy with clear therapeutic evidence of hepatotoxicity (−3), reaction published in product characteristics (+2); non-drug causes excluded the following: excluded (+2), re-administration reaction—not performed (0) with a total score of 6 indicating “probable” (6 to 8 “probable”).

Furthermore, the guidelines for diagnosing drug-induced liver injury establish laboratory threshold criteria that must be met after ruling out other non-drug-related causes. This includes an ALT level that exceeds five times the upper limit of normal (ULN) or an ALT level more significant than three times the ULN, accompanied by a simultaneous doubling of total bilirubin [36]. In our case, it was 13.29 (ALT = 784 UI/dL and ULN = 59), another piece of evidence supporting the diagnosis of DILI. Moreover, this is the same as the literature that shows a female predominance in DILI [39].

DILI is a diagnosis that requires the exclusion of alternative diagnoses, and according to current EASL Clinical Practice Guidelines [36], some liver imaging is usually performed during the diagnostic evaluation of patients suspected of it. Thus, computed tomography was performed according to the guideline recommendation (grade B recommendation) [36], which can be performed in the emergency department, successfully excluding biliary disease and other possible causes. In patients who presented with amoxicillin-clavulanic acid-related DILI, changes in both liver parenchyma and biliary tree morphology were observed [37]. In addition, abdominal discomfort may arise in cases of drug-induced cholestatic hepatitis; however, if this symptom is the most prominent, alternative causes like choledocholithiasis or ductal obstruction must be considered and eliminated through ultrasound and CT scans of the abdomen. Also, bland cholestasis can be linked to our patient’s sepsis [40]. Patients recovering after DILI usually undergo a process of gradual healing of the liver lesions, both clinically and biochemically [7], which is particularly prevalent in those with cholestatic liver lesions—an event reflected in our patient, as illustrated in Figure 2.

Due to these factors, our case report represents a unique case. Two rare diagnoses in the ED are very important to recognize due to their high morbidity and mortality [26,41]. We hope our own experiences and those of others will raise awareness among emergency department doctors about this rare but important diagnosis. With advancements in diagnostic tools and techniques, we hope that more cases of iliopsoas abscess and DILI will be accurately diagnosed.

## 4. Conclusions

The case presented here exemplifies how psoas abscess, a rare cause of back pain, can quickly go unrecognized, especially in the emergency department. It is crucial for emergency physicians to be knowledgeable about the signs and symptoms that may indicate a psoas abscess and to initiate further investigation. In patients with known risk factors for it that present the ED with fever, limp, and back pain, psoas abscess should be considered a potential cause of low back pain. Secondly, this case also documents a DILI association. The use of multidrug-induced hepatotoxicity in this patient resulted in increased AST, ALT, and BT consistent with mixed hepatocellular and cholestatic liver injury. The pharmacokinetic characteristics of all these drugs, the temporal correlation, and the positive RUCAM score indicate that this patient developed DILI.

In summary, this case report highlights that healthcare providers should take a complete history that includes accurate medication use and clinical symptoms for both diagnoses. While laboratory tests and ultrasound can provide helpful information, they alone cannot confirm or rule out the diagnosis. Therefore, superior imaging using CT or MRI is the preferred diagnostic tool.

## Figures and Tables

**Table 1:** Documented origins of iliopsoas abscess.

Primary	Secondary
❖ Idiopathic [8]	❖ Gastrointestinal: Appendicitis [9], Diverticulitis [10], Colorectal carcinoma [11], Crohn’s disease [12]
❖ Hematogenous or lymphatic seeding from a remote location [13]	❖ Reno-genital: Nephrolithiasis and Pyelonephritis [14], Urinary Tract Infection [15], Brenner tumor [16]
	❖ Musculo-Skeletal: Tuberculous Spondylodiscitis [17], Vertebral Osteomyelitis [18], Septic arthritis [19], Pott’s disease [20]
	❖ Vascular: infected aortic aneurysm [21] ❖ Skin: *ecthyma* gangrenosum [22] ❖ Other: penetrating trauma [23]

## Data Availability

The datasets are not publicly available, but Popa Daian may provide de-identified data upon request.
